# Getting Costs Right in Global Surgery

**DOI:** 10.1007/s00268-021-06300-3

**Published:** 2021-08-26

**Authors:** Mark G. Shrime

**Affiliations:** 1 Institute of Global Surgery, Royal College of Surgeons in Ireland, Dublin, Ireland; 2 Department of Global Health and Social Medicine, Harvard Medical School, Boston, USA

In 2015, my colleagues and I estimated that over 80 million people each year experience financial catastrophe due to the costs of getting surgery [1].

This estimate and the resulting World Bank annual updates are the result of a model built on the best available evidence at the time. However, as George Box famously wrote, “All models are wrong, but some are useful,” [2] a fact that is no less true of our catastrophic expenditure model. The biggest data deficit we face has been for out-of-pocket surgical costs, especially in countries in the global South [3].

This has begun to change, if slowly. Since 2015, 72 papers addressing the cost of surgery in lower-resource populations have been indexed on PubMed. The recent paper by Umo and James adds to this evidence [4]. The authors find that the total cost of surgery for an acute appendix in Papua New Guinea, including both hospital- and patient-level costs, ranges from US$11,300 and US$13,300. Of that, patients’ hospital fees amount to only $124. (For context, the per capita gross domestic product in the country is US$2636 per year [5].)

This speaks volumes. First, it highlights the intricacies of costing. The authors chose a rather unconventional hospital-plus-patient perspective to their costing study. In doing so, however, they present an almost-complete picture of the overall cost the health system bears to deliver an appendectomy.

Choosing the correct perspective is important. When cost-effectiveness studies are undertaken, a societal costing perspective like the authors’ is appropriate: outcomes like disability- and quality-adjusted life years are societal, so the costing must follow suit. On the other hands, studies of non-medical outcomes of surgical care, such as financial catastrophe, require a patient-level costing to be disentangled from the societal cost.

This also speaks volumes about the necessity of costing data to confirm or reject modeled estimates. In this case, the model is in line with what the authors find: The World Bank estimates that 3.8% of Papua New Guineas would face catastrophic expenditure if they needed surgery [5]. Using Umo and James’s out-of-pocket costs, the estimate rises to 5.8% of the population of Papua New Guinea, with a 95% uncertainty interval (1.7–12.4%) that includes the original.

With time, even the best models become less relevant. The risk of financial catastrophe due to surgery does not. Ongoing studies into patient- and system-level costs for surgery continue to be an imperative.
